# The relationship between compartment models and their stochastic counterparts: A comparative study with examples of the COVID-19 epidemic modeling

**DOI:** 10.7555/JBR.37.20230137

**Published:** 2024-03-05

**Authors:** Ziyu Zhao, Yi Zhou, Jinxing Guan, Yan Yan, Jing Zhao, Zhihang Peng, Feng Chen, Yang Zhao, Fang Shao

**Affiliations:** 1 Department of Biostatistics, School of Public Health, Nanjing Medical University, Nanjing, Jiangsu 211166, China; 2 Nanjing Hanwei Public Health Research Institute Co., Ltd, Nanjing, Jiangsu 210005, China; 3 China International Cooperation Center for Environment and Human Health, Center for Global Health, Nanjing Medical University, Nanjing, Jiangsu 211166, China; 4 The Center of Biomedical Big Data and the Laboratory of Biomedical Big Data, Nanjing Medical University, Nanjing, Jiangsu 211166, China; 5 Jiangsu Key Lab of Cancer Biomarkers, Prevention and Treatment, Collaborative Innovation Center for Cancer Personalized Medicine, Nanjing Medical University, Nanjing, Jiangsu 211166, China

**Keywords:** compartment models, agent-based models, compartment-agent mixed models, comparative study, COVID-19

## Abstract

Deterministic compartment models (CMs) and stochastic models, including stochastic CMs and agent-based models, are widely utilized in epidemic modeling. However, the relationship between CMs and their corresponding stochastic models is not well understood. The present study aimed to address this gap by conducting a comparative study using the susceptible, exposed, infectious, and recovered (SEIR) model and its extended CMs from the coronavirus disease 2019 modeling literature. We demonstrated the equivalence of the numerical solution of CMs using the Euler scheme and their stochastic counterparts through theoretical analysis and simulations. Based on this equivalence, we proposed an efficient model calibration method that could replicate the exact solution of CMs in the corresponding stochastic models through parameter adjustment. The advancement in calibration techniques enhanced the accuracy of stochastic modeling in capturing the dynamics of epidemics. However, it should be noted that discrete-time stochastic models cannot perfectly reproduce the exact solution of continuous-time CMs. Additionally, we proposed a new stochastic compartment and agent mixed model as an alternative to agent-based models for large-scale population simulations with a limited number of agents. This model offered a balance between computational efficiency and accuracy. The results of this research contributed to the comparison and unification of deterministic CMs and stochastic models in epidemic modeling. Furthermore, the results had implications for the development of hybrid models that integrated the strengths of both frameworks. Overall, the present study has provided valuable epidemic modeling techniques and their practical applications for understanding and controlling the spread of infectious diseases.

## Introduction

In December 2019, a novel enveloped RNA beta-coronavirus that causes coronavirus disease 2019 (COVID-19), emerged in Wuhan, China^[1]^. Over the past few years, the COVID-19 pandemic had significant impacts on the global economy, society, and public health, including school closures, industry collapses, and millions of job losses^[2]^. There are individual data-based approaches to modeling and analyzing the course of COVID-19 infections^[3–4]^, and macro data-based epidemiological modeling approaches to interpreting and controlling the spread of COVID-19. Compartment models (CMs) and agent (individual)-based models (ABMs) are two representative frameworks to investigate the dynamics of epidemics and the efficiency of prevention strategies^[5]^. Although systematic comparisons of these two models have been made available in the literature^[6–9]^, the relationship between deterministic CM and its corresponding stochastic version of ABM has not been well studied. This knowledge gap hinders the ability to effectively compare and integrate these modeling approaches.

CM is a classic approach to epidemic modeling that can be traced back to about 100 years ago^[10–11]^. Classical CMs are continuous-time dynamic systems based on nonlinear differential equations that are conventionally solved by numerical methods. CMs assume homogeneous mixing within populations and are computationally efficient, but may not accurately capture individual-level behavior and transmission rates of heterogeneous disease^[6]^. In infectious disease epidemiology, the SEIR model^[12]^ is one of the most well-known CM, in which the population is divided into susceptible (S), exposed (E), infectious (I), and recovered (R) compartments. The SEIR model is an extension of the classic SIR model^[11]^. The SEIR model and its extensions have been widely used in the studies of COVID-19 pandemic^[12–20]^.

Stochastic compartment models (SCMs) have been developed to address the limitations of CMs by incorporating stochastic events and discrete-time transitions between compartments^[21–23]^. It provides a more accurate representation of individual-level behavior and avoids the assumption of homogeneous mixing. SCMs have been widely used in the study of infectious diseases^[24]^. Similar to CMs, SCMs are computationally efficient but may vary wildly because of the variances of random variables.

ABM is a relatively new stochastic approach to modeling complex systems by representing individual agents with their characteristics and interactions. Agents represent individuals, households, governments, or any other entities of interest, and adapt their behaviors in response to interactions with other agents and their environment. The use of ABMs in public health has been advocated by Rutter *et al*^[25]^, and the use of ABMs for COVID-19 epidemic modeling has also been proposed in Australia, Luxembourg, and Switzerland^[26–28]^.

CM is a top-down modeling method, while ABM is a bottom-up modeling method. ABM is based on many individual agents with their own actions and the ability to interact with each other, while CM models subpopulations of different states as a few compartments. Therefore, CM is much less computationally intensive than ABM. On the other hand, ABM is more versatile and flexible than CM. ABM easily involves the spatial movement of agents, but CM requires a dynamic system with much more complicated partial differential equations than ordinary differential equations to achieve this. CM can be converted to its corresponding ABM like SCM^[26]^, but ABM for a complex system may not have its CM counterpart.

The similarities between CMs, SCMs, and ABMs have been noted by many investigators^[7–9]^. However, there is a lack of comprehensive understanding regarding the relationship between CMs and their corresponding stochastic models, which hinders the ability to effectively compare and integrate these modeling approaches. This gap motivated us to conduct a comparative study of CM and its corresponding stochastic models to illustrate the relationship among them. In the remainder of this manuscript, we presented the relationship with the selected CMs by theory and simulation. Three representative CMs (SEIR, susceptible, exposed, infected, recovered and deceased [SEIRD], and susceptible, exposed, infected, hospitalized and removed [SEIHR]) in COVID-19 pandemic studies^[17–19]^ were considered. By establishing the equivalence between numerical solutions of CMs using the Euler scheme^[29]^ and their stochastic counterparts, we aimed to enhance our understanding of the relationships among these models. Furthermore, we acknowledged the limitations of discrete-time stochastic models in perfectly reproducing the exact solutions of continuous-time CMs. To overcome this challenge, we proposed an efficient model-calibration method allowing the replication of CM exact solutions in corresponding stochastic models through parameter adjustment, which minimized the differences between the CM exact solutions and CM numerical solutions by the Euler scheme. Additionally, we introduced a novel stochastic compartment-agent mixed model (CAMM) as an alternative approach to ABM, which offered a promising solution for conducting large-scale population simulations with a limited number of agents. By bridging the gap between deterministic CMs and stochastic models, we explored advanced epidemic modeling to facilitate the comparison, unification, and hybridization of these modeling approaches, ultimately improving our ability to understand and control the spread of infectious diseases.

## Materials and methods

### Models

The SEIR model and its extensions are classical CMs widely used in the COVID-19 pandemic studies^[12–20]^. These extended models incorporated more compartments^[16,18–20]^ and the effects of important factors, such as vaccination and social distance^[20,30–34]^, government action and public response^[35–36]^, media^[37–38]^, and environments^[34,36,39]^. In the present study, the SEIR model and its selected extensions were presented as typical representative CMs for comparisons with their corresponding stochastic models to demonstrate the relationships among them with more details shown afterward.

CMs are conventionally solved by numerical methods. In the present study, numerical solutions by the Euler scheme^[29]^ were considered for comparisons. However, the Euler scheme, which is also known as the first-order Runge-Kutta method, is the most simple and basic numerical scheme, and cannot derive exact solutions in many situations. Therefore, numerical solutions by more sophisticated methods, such as LSODA^[40]^ and the fourth-order Runge-Kutta^[29]^, are considered exact solutions of CMs for comparisons.

In the literature^[8,24]^, CMs were converted to their corresponding SCMs and ABMs using Bernoulli, binomial, and multinomial distributed random variables. Solutions of SCMs and ABMs can be verified to be equivalent to numerical solutions of the corresponding CMs by the Euler scheme.

While CMs and ABMs had their advantages and disadvantages, some investigators proposed hybrid models (HMs) that combined both approaches^[41]^. HMs can switch between CM and ABM under certain conditions to balance computational efficiency and modeling accuracy.

Because of the limitation of computation resources, it is difficult for ABMs to simulate the activity of individuals in a large population. This is because ABMs require more computational power and become computationally expensive as the population size grows. HM can be treated as a solution to this issue by switching ABM to CM, when the number of agents exceeds the maximum number that can be simulated. However, HM is a bridge between CM and ABM, and it is neither a purely deterministic nor a purely stochastic model. To compare CMs with their stochastic counterparts, a novel model, the CAMM, was proposed as an alternative to ABM to simulate large populations with a limited number of agents in the present study. Different from HM, CAMM directly models compartments as agents under the ABM framework, and merges agents with the same state, when the number exceeds a predefined maximum, which allows for the efficient modeling of complex systems with large populations under the ABM framework with more details shown afterward.

In the present study, CMs were compared with their stochastic counterparts, including SCMs, ABMs, and CAMMs, which were proposed by the authors and illustrated in the following sections. Four scenarios with three compartment models (SEIR, SEIRD, and SEIHR) and their stochastic counterparts are as follows.

### Scenarios 1 and 2: SEIR model

**Figure 1:**
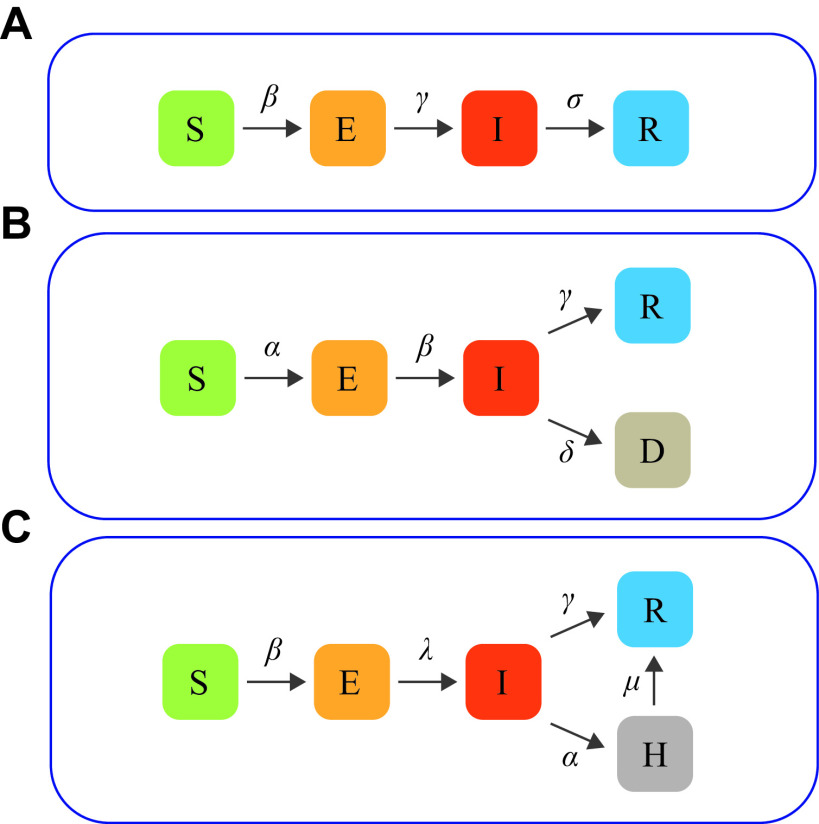
Illustrations of the compartmental models.

The SEIR model was utilized in a study by Zhou and Liu *et al*^[17]^, in which they estimated the basic reproduction number of COVID-19 in Wuhan by using the SEIR model, thus concluding that the early transmission of COVID-19 was close to, or slightly higher than, that of severe acute respiratory syndrome (SARS). The SEIR model divided the population into four compartments (***Fig. 1A***): susceptible (S), exposed (E), infected (I), and recovered (R). It can be expressed by the following nonlinear system of ordinary differential equations.

1 \begin{equation*}\begin{split} 
\left\{ {\begin{array}{*{20}{l}}
 {\dfrac{{dS(t)}}{{dt}} = - \beta \dfrac{{I(t)}}{N}S(t)\;\;\;{\kern 1pt} \quad \;} \\ 
 {\dfrac{{dE(t)}}{{dt}} = \beta \dfrac{{I(t)}}{N}S(t) - \gamma E(t)} \\ 
 {\dfrac{{dI(t)}}{{dt}} = \gamma E(t) - \sigma I(t)\;\;\;{\kern 1pt} \;\;\;{\kern 1pt} } \\ 
 {\dfrac{{dR(t)}}{{dt}} = \sigma I(t)\;\;\;{\kern 1pt} \;\;\;{\kern 1pt} \;\;\;{\kern 1pt} \;\;\;{\kern 1pt} \;\;\;{\kern 1pt} } 
\end{array}} \right. 
\end{split}\end{equation*}

$S$ is the number of susceptible individuals; $E$ is the number of individuals exposed (infected but not yet contagious); $I$ is the number of individuals infected (contagious); $R$ is the number of individuals recovered (immune); $N$ is the total population size and $N = S\left( t \right) + E\left( t \right) + I\left( t \right) + R\left( t \right)$; $\beta $ is the transmission rate (the rate at which susceptible individuals become exposed); $\gamma $ is the incubation rate (the rate at which exposed individuals become infected); and $\sigma $ is the recovery rate (the rate at which infected individuals recover and gain immunity).

The basic reproduction number, $ {R_0} $, is a vital metric in the field of infectious diseases, as it indicates the level of contagion^[17,42–43]^. The epidemiological definition of $ {R_0} $ refers to the average number of secondary cases generated by a single infected individual in a population entirely of susceptible individuals^[42]^. The infectious disease can evolve into an epidemic only if $ {R_0} > 1 $. For the SEIR model, $ {R_0} = \dfrac{{\beta S \left( 0 \right)}}{{\gamma N}} $.

The SEIR model is a set of four ordinary differential equations that describe how the number of individuals in each compartment changes over time. The equations are based on the assumption that the population is closed, meaning that there is no migration of individuals into or out of the population during the study period.

Differential equations can be solved by numerical methods. The following discrete-time model is equivalent to the basic Euler numerical scheme (the Euler method or the first-order Runge-Kutta method^[29]^) for solving the SIER model. If we set equally spaced discrete time step $t \in \left\{ {0, \;1, \;2,\; \cdots ,\; T - 1} \right\}$ with the time span $[0,T]$ for simplicity, then

2 \begin{equation*}\begin{split} 
\begin{array}{c}\left\{\begin{array}{l}{\hat{S}}_{t+1}={\hat{S}}_{t}-\dfrac{\beta {\hat{S}}_{t}{\hat{I}}_{t}}{N}             \\ {\hat{E}}_{t+1}={\hat{E}}_{t}+\dfrac{\beta {\hat{S}}_{t}{\hat{I}}_{t}}{N}-\gamma {\hat{E}}_{t} \\ {\hat{I}}_{t+1}={\hat{I}}_{t}+\gamma {\hat{E}}_{t}-\sigma {\hat{I}}_{t}      \\ {\hat{R}}_{t+1}={\hat{R}}_{t}+\sigma {\hat{I}}_{t}  \end{array}\right. \end{array}
\end{split}\end{equation*}

${\hat S_t}$ stands for the estimated number of susceptible individuals at the discrete time point $ t $, *i.e.*, ${\hat S_t} = \hat S\left( t \right)$, in the same manner as other states, and $ [ {{{\hat S}_0},\;{{\hat E}_0},\;{{\hat I}_0},\;{{\hat R}_0}} ] = [ {S \left( 0 \right),\;E\left( 0 \right),\;I\left( 0 \right),\;R\left( 0 \right)} ] $.

It is important to note that the numerical solution of ordinary differential equations using the above Euler method is different from the exact solution and the numerical solutions by other more sophisticated and accurate numerical schemes, such as Runge-Kutta or LSODA, in general.

Based on the above Euler scheme, the corresponding SCM can be derived as follows^[8]^.

3 \begin{equation*}\begin{split} 
\left\{ {\begin{array}{*{20}{l}}
 
 {{{\hat S}_{t + 1}} = {{\hat S}_t} - {s_t}} 
 \\ 
 {{{\hat E}_{t + 1}} = {{\hat E}_t} + {s_t} - {e_t}} \\ 
 {{{\hat I}_{t + 1}} = {{\hat I}_t} + {e_t} - {i_t}} \\ 
 {{{\hat R}_{t + 1}} = {{\hat R}_t} + {i_t}} \\ 
 {{s_t}\sim Binomial\left( {{{\hat S}_t},\;\dfrac{{\beta {{\hat I}_t}}}{N}} \right)} \\ 
 {{e_t}\sim Binomial\left( {{{\hat E}_t},\;\gamma } \right)} \\ 
 {{i_t}\sim Binomial\left( {{{\hat I}_t},\;\sigma } \right)} 
\end{array}} \right.
\end{split}\end{equation*}

In SCM, individuals in a compartment except *R* will be transformed into the next compartment with a certain probability, which is the same as the rate of change in CM.

The corresponding ABM is as follows.

4 \begin{equation*}\begin{split} 

 {{a_i}(t + 1) = \left\{  \begin{array}{*{20}{l}}


 {{a_i}(t) + Bernoulli\left( {\dfrac{{\beta {{\hat I}_t}}}{N}} \right),\;{\text{ if }}{a_i}(t) = 1} \\ 
 {{a_i}(t) + Bernoulli\left( \gamma \right),\;\quad {\text{ if }}{a_i}(t) = 2} \\ 
 {{a_i}(t) + Bernoulli\left( \sigma \right),\,\quad {\text{ if }}{a_i}(t) = 3} 
  \\
 {a_i}(t),{\text{ }}\;\;\;{\kern 1pt} \;\;\;\;\;{\kern 1pt} \;\;\;\;\;\;{\kern 1pt} \;\,\;{\kern 1pt} \qquad \quad  {\text{ if }}{a_i}(t) = 4  \\ 

\end{array}
 \right.} \end{split}\end{equation*}

Here, ${a_i}\left( t \right)$ is the agent $i \in \{ 1,\; 2,\; \cdots ,\; N\} $ at the time $t \in \{ 0, \;1, \;\cdots , \;T\} $, and the values of ${a_i}\left( t \right)$, 1, 2, 3, and 4 refer to the *S*, *E*, *I*, and *R* compartment States, respectively. ${\hat I_t}$ is the number of agents in State *I* at the time $t$.

When the population size *N* is large, the number of agents is also *N*, which makes the simulation of ABM intractable. Therefore, to overcome this difficulty, we proposed to combine compartments and agents to construct a CAMM with the advantages of both CM and ABM. Compartments are treated as union sets of agents with states and sizes, and agents in the same state can be concatenated to a compartment by summing up their sizes. In this manner, ABM-type simulations can be implemented with a predefined limited maximum number of agents. The algorithm for CAMM is shown as follows.

For the time step $t = 0$, the initial step is performed as follows.

**Step 0**. Generate only four agents, $ {a}_{i,\;0} $, where $S tate\left( {{a_{i,\;0}}} \right) = i$ and $S ize\left( {{a_{i,\;0}}} \right)$ is the *i*-th element of $ \left[ {S \left( 0 \right),\;E\left( 0 \right),\;I\left( 0 \right),\;R\left( 0 \right)} \right] $ for $i \in \left\{ {1,\;2,\;3,\;4} \right\}$. Here, the values of $S tate\left( {{a_{i,\;0}}} \right)$, 1, 2, 3, and 4, refer to the *S*, *E*, *I*, and *R* compartment states, respectively. $S ize\left( {{a_{i,\;0}}} \right)$ is the size of the compartment agent with nonnegative integer values.

For each discrete time step $t \in \left\{ {0,\;1,\;2,\; \cdots ,\;T  -  1} \right\}$, the following two steps are performed.

**Step 1**. For each agent ${a_{i,\;t}}$ at a time step $t$, $S tate\left( {{a_{i,\;t + 1}}} \right) = S tate\left( {{a_{i,\;t}}} \right)$ and $S ize\left( {{a_{i,\;t + 1}}} \right) = S ize\left( {{a_{i,\;t}}} \right) -   a{s_{i,\;t}}$.

Next, if $S tate\left( {{a_{i,\;t}}} \right) < 4$, a new agent ${a_{new\_i,\;t + 1}}$ is generated with $ S tate\left( {{a_{new\_i,\;t + 1}}} \right) = S tate\left( {{a_{i,\;t}}} \right) + 1 $ and $S ize\left( {{a_{new\_i,\;t + 1}}} \right) = a{s_{i,\;t}}$,

5 \begin{equation*}\begin{split} 
    \begin{array}{*{20}{l}}
 {a{s_{i,\;t}}\sim \left\{ {\begin{array}{*{20}{l}}
 {Binomial\left( {{S ia_{i,\;t}},\;\dfrac{{\beta {{\hat I}_t}}}{N}} \right),\;\;{\text{ if }}S tate\left( {{a_{i,\;t}}} \right) = 1} \\ 
 {Binomial\left( {{S ia_{i,\;t}},\;\gamma } \right),\;\,\quad{\text{ if }}S tate\left( {{a_{i,\;t}}} \right) = 2} \\ 
 \begin{gathered}
 Binomial\left( {{S ia_{i,\;t}},\;\sigma } \right),\,\quad{\text{ if }}S tate\left( {{a_{i,\;t}}} \right) = 3  \\
 0,\qquad \qquad\qquad\quad\quad\;\;\;{\text{ if }}S tate\left( {{a_{i,\;t}}} \right) = 4   
\end{gathered} 
\end{array}} \right.} 
\end{array}
\end{split}\end{equation*}

Here, the values of $S tate\left( {{a_{i,\;t}}} \right)$, 1, 2, 3, and 4, refer to the *S*, *E*, *I*, and *R* compartment states, respectively. $S i{a_{i,\;t}} = S ize\left( {{a_{i,\;t}}} \right)$ is the size of the compartment agent with nonnegative integer values. ${\hat I_t}  =   \mathop \sum \limits_{S tate\left( {{a_{j,\;t}}} \right) = 3} S ize\left( {{a_{j,\;t}}} \right)$ is the total number of individuals with state *I* at the time $t$.

**Step 2**. If the total number of agents at the time step $t + 1$ exceeds the predefined maximum number of agents, agents are concatenated to *S*, *E*, *I*, and *R* compartments as follows. For $i \in \left\{ {1,\;2,\;3,\;4} \right\}$,

$S tate\left( {{a_{i,\;t + 1}}} \right)  =  i$ and $ S ize\left( {{a_{i,\;t + 1}}} \right)  =   \mathop \sum \limits_{S tate\left( {{a_{j,\;t + 1}}} \right)  =  i}  $$S ize\left( {{a_{j,\;t + 1}}} \right) $.

Remove all other agents ${a_{i,\;t + 1}}$ for $i \notin \left\{ {1,\;2,\;3,\;4} \right\}$.

CAMM combined the advantages of both CM and ABM with high efficiency. CAMM was somewhat similar to HM, which switched between CM and ABM. However, unlike HM, CAMM directly treated compartments as agents and modeled them under the ABM framework. CAMM was proposed as an alternative modeling method to ABM for large population simulations with a limited number of agents.

### Scenario 3: SEIRD model

Many epidemiological models are derived from SEIR. In this scenario, we used a SEIRD model to compare CM, SCM, ABM, and CAMM. The model was from Shin *et al*^[18]^, who analyzed the time-varying transmission dynamics of the COVID-19 epidemic in Republic of Korea over multiple stages of development, demonstrating that the model offered a better model fit and could show how the infection pattern of COVID-19 changes over time. The SEIRD model divided the population into five compartments (***Fig. 1B***): susceptible (S), exposed (E), infected (I), recovered (R), and deceased (D).

6 \begin{equation*}\begin{split} 
\begin{array}{c}\left\{\begin{array}{l}\dfrac{dS\left(t\right)}{dt}=-\dfrac{\alpha I\left(t\right)}{N}S\left(t\right)                    \\ \dfrac{dE\left(t\right)}{dt}=\dfrac{\alpha I\left(t\right)}{N}S\left(t\right)-\beta E\left(t\right)        \\ \dfrac{dI\left(t\right)}{dt}=\beta E\left(t\right)-\gamma I\left(t\right)-\delta I\left(t\right) \\ \dfrac{dR\left(t\right)}{dt}=\gamma I\left(t\right)                                  \\ \dfrac{dD\left(t\right)}{dt}=\delta I\left(t\right)                                  \end{array}\right.\end{array}
\end{split}\end{equation*}

The usage of each parameter in its original study was retained, which differed slightly from the SEIR model. $N$ is the total population size, $N = S\left( t \right) + E\left( t \right) + I\left( t \right) + R\left( t \right) + D\left( t \right)$, $\alpha $ is the transmission rate (the rate at which susceptible individuals become exposed), $\beta $ is the incubation rate (the rate at which exposed individuals become infected), $\gamma $ is the recovery rate (the rate at which infected individuals recover and become immune), and $\delta $ is the fatality rate (the rate at which infected individuals die). For the SEIRD model, $ {R_0} = \dfrac{{\alpha S \left( 0 \right)}}{{\left( {\gamma + \delta } \right)N}} $.

Our comparison involved the CM, SCM, ABM, and CAMM. Corresponding stochastic models of SEIRD were derived using the same method as scenarios 1 and 2. Details are shown in ***Supplementary Section 1.1***, available online.

### Scenario 4: SEIHR model

The SEIHR model is another extension of the SEIR model. It was proposed by Wang *et al*^[19]^ to study the COVID-19 epidemic in Wuhan after the blockade, in the case of no population inflow or outflow and certain control of COVID-19 in China. We cited this SEIHR model to compare CM, SCM, and CAMM.

The SEIHR model divided the population into five compartments (***Fig. 1C***): susceptible (S), exposed (E), infected (I), hospitalized (H), and removed (R). The infected class included symptomatic and asymptomatic infections, and the removed class included individuals who recovered and deceased, and excluded natural births and deaths. In this model, a hospitalized class for the number of daily hospitalizations was introduced, which were obtained from public data. When a state change occurred in the infected compartment, individuals who have been confirmed were transferred to the hospitalized state while those who have not been confirmed were transferred to the removed state. The SEIHR model can be represented by the following nonlinear ordinary differential equations.

7 \begin{equation*}\begin{split} 
 \left\{ {\begin{array}{*{20}{l}}
 {\dfrac{{dS(t)}}{{dt}} = - \beta \left[ {1 - \dfrac{{I(t)}}{N}} \right]\dfrac{{I(t)}}{N}S(t)} \\ 
 {\dfrac{{dE(t)}}{{dt}} = \beta \left[ {1 - \dfrac{{I(t)}}{N}} \right]\dfrac{{I(t)}}{N}S(t) - \lambda E(t)} \\ 
 {\dfrac{{dI(t)}}{{dt}} = \lambda E(t) - \alpha I(t) - \gamma I(t)} \\ 
 {\dfrac{{dH(t)}}{{dt}} = \alpha I(t) - \mu H(t)} \\ 
 {\dfrac{{dR(t)}}{{dt}} = \gamma I(t) + \mu H(t)} 
\end{array}} \right.
\end{split}\end{equation*}

The parameters also differed slightly from the SEIR model. $N$ is the total population size, $N = S\left( t \right) +   E\left( t \right) + I\left( t \right) + H\left( t \right) + R\left( t \right)$; $\,\;\beta \left[ {1 - \dfrac{{I(t)}}{N}} \right]$ indicated the daily rate of progression from susceptible status to exposed status; $\lambda $ is the daily rate of progression from exposed status to infected status; $ \alpha $ is the daily rate of progression from infected status to hospitalized status; $ \gamma $ is the daily rate of progression from infected status to removed status; $\mu $ is the daily rate of progression from hospitalized status to removed status. For the SEIHR model, $ {R_0} = \dfrac{{\beta S \left( 0 \right)}}{{\left( {\alpha + \gamma } \right)N}} $.

In this scenario, CM, SCM, and CAMM were used to simulate the population change. ABM was omitted in this scenario because of the large population size *N*. Corresponding stochastic models of SEIHR were derived using the same method as scenarios 1 and 2. Details are shown in ***Supplementary Section 1.2***, available online.

### Equivalence of models and model calibration

Gallagher^[8]^ has proved the equivalence of the numerical solution of CM by the Euler method, the corresponding SCM, and ABM, *i.e.,* the expected value of the corresponding SCM and the expected value of the corresponding ABM in terms of the state sizes at each time step was unbiased with the solution of the Euler method. However, the numerical solution by the Euler scheme was confused with the exact solution in the proofs of her thesis^[8]^. The equivalence of CM solution by the Euler method as well as the corresponding SCM, ABM, and CAMM can also be verified in the same manner.

However, as mentioned before, the numerical solutions by other sophisticated methods were different from those by the Euler method. Therefore, to mimic the solutions by other numerical methods with stochastic counterparts, it was first necessary to calibrate SCM, ABM, and CAMM. However, directly estimating the parameters to fit SCM, ABM, and CAMM to exact numerical solutions was time-consuming because of the calculation of means for SCM, ABM, and CAMM replication results as stochastic model estimates. Therefore, we proposed to estimate parameters using the numerical solutions by the Euler method instead of those of SCM, ABM, and CAMM because of their equivalence. This calibration strategy was much more efficient than the traditional estimation method.

### Comparative analysis

CM and its stochastic counterparts, including SCM, ABM, and CAMM, were compared for three CMs (SEIR, SEIRD, and SEIHR) in four scenarios. Scenarios 1 and 2 were SEIR models with different initial values. Scenarios 3 and 4 were for SEIRD and SEIHR models, respectively. The parameter settings of these models were derived from the results of the corresponding literature for these models^[17–19]^, respectively.

For stochastic models including SCM, ABM, and CAMM, repeated simulations were performed. The estimates for states were calculated as the means of the replications of the stochastic models at each time point and state. One thousand replications were set for simulations. The exact solutions of CM were calculated by the LSODA numerical scheme, which was the default solver for the R package "deSolve"^[44]^ for numerical solutions of CM. To compare the results of CM, SCM, ABM, and CAMM, the exact solution of CM was treated as the benchmark to obtain the root mean squared error (RMSE) and mean absolute error (MAE) of the calculated state percentages of the initial population *N* by other methods. Parameter estimations for model calibration were also implemented to minimize the RMSE of the calculated state percentages of the initial population.

All statistical analyses were performed using R software^[45]^, version 4.2.2 with R package "deSolve" (version 1.34)^[44]^. Sample codes for simulations and analyses in the present study are provided with details in ***Supplementary Section 2***, available online.

## Results

The results of the proposed three CMs and their stochastic counterparts in four scenarios were demonstrated in this section.

### Scenarios 1 and 2: SEIR model

The SEIR model was constructed based on the "Materials and methods" section. The equivalence of CM using the Euler method, the corresponding SCM, ABM, and CAMM, and the difference between the CM exact solution by LSODA and the former ones were demonstrated by simulations. Model calibration was conducted to adjust the parameter values of the stochastic methods so that their solutions were as consistent as possible with the CM solutions by LSODA.

The formulas for CM were the same as the above with derived parameters $\left( {\beta ,\;\gamma ,\;\sigma } \right) =    \left( {\dfrac{{2.83}}{{2.94}},\;\dfrac{1}{{5.46}},\;\dfrac{1}{{2.94}}} \right)$^[17–19]^. The initial values of the model were set to $\left[ {S(0),\;E(0),\;I(0),\;R(0)} \right] = \left[ {1\,000,\;0,\;10,\;0} \right]$ and $N = 1\,010$ with the simulation time span $[0,\;T] = [0,\;50]$ and $ {R_0} = \dfrac{{\beta S \left( 0 \right)}}{{\gamma N}} = 5.203\,7 $. CM solutions by the LSODA (CM) and Euler methods (Euler) as well as the corresponding SCM, ABM, and CAMM shared the same parameters. The simulation replication number for SCM, ABM, and CAMM, and the maximum number of agents for CAMM were set to 1000. RMSE and MAE were shown to evaluate the model effect.

Based on RMSEs and MAEs in scenario 1 (***Table 1***), the Euler method solutions were close to those of SCM, ABM, and CAMM, which demonstrated their equivalence. However, RMSE_0_ and MAE_0_ were larger than RMSE_1_ and MAE_1_, respectively. Therefore, model calibration was needed to mimic the CM solutions by other sophisticated numerical methods using the Euler method, SCM, ABM, and CAMM. Model calibration for these four methods was conducted as stated before, and the calibrated parameters were efficiently obtained using the Euler method solutions, which were $(\beta ,\;\gamma ,\;\sigma ) = (1.091\;4,  0.184\;3,\;0.400\;8)$ in this scenario. After calibration, RMSE_0_ and MAE_0_ decreased significantly, compared with the results before calibration, and RMSE_1_ and MAE_1_ remained at the same level. The solutions of Euler, SCM, ABM, and CAMM were close to the solution by the LSODA method, showing the effectiveness of our proposed calibration. However, even after calibration, the difference between numerical solutions of CM by the LSODA and Euler methods remained, which may be because of the inherent accuracy difference between the LSODA and Euler methods. Therefore, the corresponding discrete-time stochastic models cannot perfectly reproduce the exact solution of continuous-time CM.

**Table 1:** RMSE and MAE of CM by the Euler method, SCM, and CAMM before and after model calibration

Scenarios	Model	Before calibration					After calibration			
		RMSE_0_	MAE_0_	RMSE_1_	MAE_1_		RMSE_0_	MAE_0_	RMSE_1_	MAE_1_
1	Euler	1.528	1.041	0.000	0.000		0.781	0.610	0.000	0.000
	SCM	1.951	1.296	0.781	0.502		1.082	0.747	0.879	0.573
	ABM	2.060	1.370	0.873	0.568		1.031	0.715	0.798	0.523
	CAMM	2.247	1.502	1.051	0.700		1.125	0.774	0.940	0.612
2	Euler	1.377	0.955	0.000	0.000		0.409	0.301	0.000	0.000
	SCM	1.377	0.955	0.001	0.001		0.408	0.300	0.003	0.002
	CAMM	1.376	0.954	0.001	0.000		0.408	0.300	0.002	0.001
3	Euler	1.132	0.637	0.000	0.000		0.418	0.305	0.000	0.000
	SCM	1.283	0.726	0.179	0.102		0.396	0.286	0.098	0.053
	ABM	1.187	0.670	0.092	0.053		0.387	0.281	0.068	0.039
	CAMM	1.188	0.671	0.095	0.052		0.410	0.291	0.145	0.081
4	Euler	0.551	0.277	0.000	0.000		0.367	0.183	0.000	0.000
	SCM	0.550	0.276	0.001	0.000		0.369	0.184	0.003	0.002
	CAMM	0.550	0.276	0.000	0.000		0.365	0.182	0.002	0.001
RMSE_0_ and MAE_0_ used the numerical solution of the CM model by the LSODA method as the benchmark, and RMSE_1_ and MAE_1_ used the numerical solution of the CM model by the Euler method as the benchmark. Scenario 1 was set to simulate the SEIR model with experiment data. Scenario 2 was set to simulate the SEIR model with real world data. Scenario 3 was set to simulate the SEIRD model with real world data. Scenario 4 was set to simulate the SEIHR model with real world data. Abbreviations: RMSE, root of mean squared error; MAE, mean absolute error; CM, compartment model; SCM, stochastic compartment model; CAMM, compartment-agent mixed model; ABM, agent-based model.										

**Figure 2:**
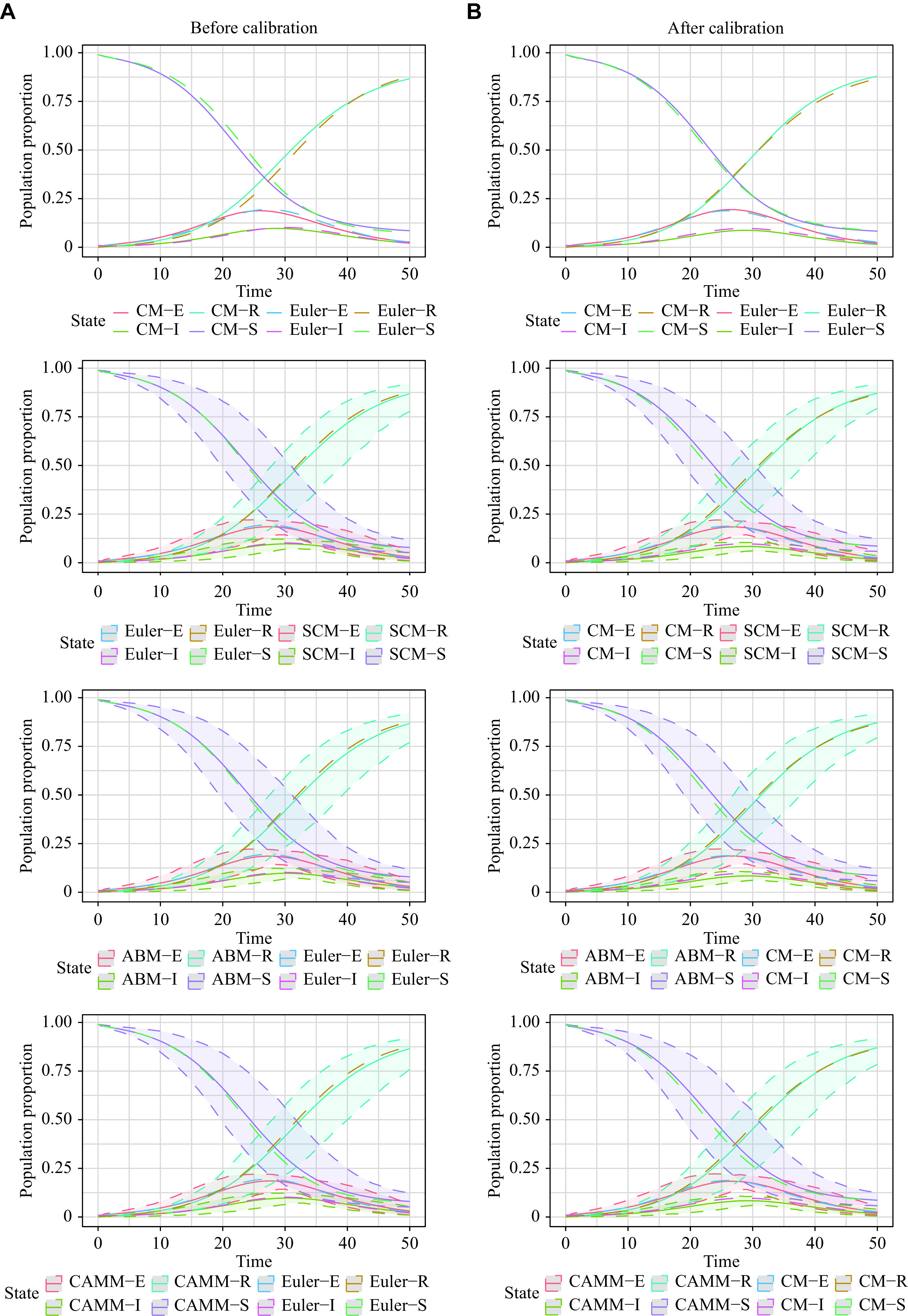
Population change curves of *S*, *E*, *I*, and *R* states of CM, SCM, ABM, and CAMM in scenario 1.

***Fig. 2*** demonstrates these results graphically. The curves of CM by the Euler method and SCM as well as CAMM almost overlapped before and after calibration, verifying the equivalence of CM by the Euler method, SCM and CAMM. However, there was a clear difference between CM by the LSODA method and CM by the Euler method before calibration, which was significantly reduced after calibration.

For scenario 2, the same SEIR model and parameters of scenario 1 were implemented with different initial values $\left[ {S \left( 0 \right),\;E\left( 0 \right),\;I\left( 0 \right),\;R\left( 0 \right)} \right] =   \left[ {12\,323\,698,\;1\,412,\;1\,408,\;0} \right]$ and $N = 12\,326\,516$^[17]^. These data were from real-world data in Wuhan. Since the total population *N* was large, the corresponding ABM was intractable for a large number of agents, *N*. CAMM was implemented as the alternative method for ABM. Therefore, CM by the LSODA method (CM), CM by the Euler method (Euler), corresponding SCM, and CAMM were compared. The simulation replication number for SCM and CAMM, and the maximum number of agents for CAMM were set to 1000. RMSE and MAE were calculated as the RMSE and MAE for *E* and *I* States' percentages of the initial population *N*. Parameters for solutions of CM by LSODA remains $\left( {\beta ,\;\gamma ,\;\sigma } \right) = \left( {\dfrac{{2.83}}{{2.94}},\;\dfrac{1}{{5.46}},\;\dfrac{1}{{2.94}}} \right)$ and $ {R_0} =   \dfrac{{\beta S \left( 0 \right)}}{{\gamma N}} = 5.254\,5 $. Model calibration for Euler, SCM, and CAMM was conducted as Stated before. Parameters for these three methods were efficiently adjusted by minimizing the RMSE_0_ of the Euler method solutions. Calibrated parameters were $(\beta ,\;\gamma ,\;\sigma ) = (1.031\,7,\;0.192\,0,\;0.365\,0)$.

**Figure 3:**
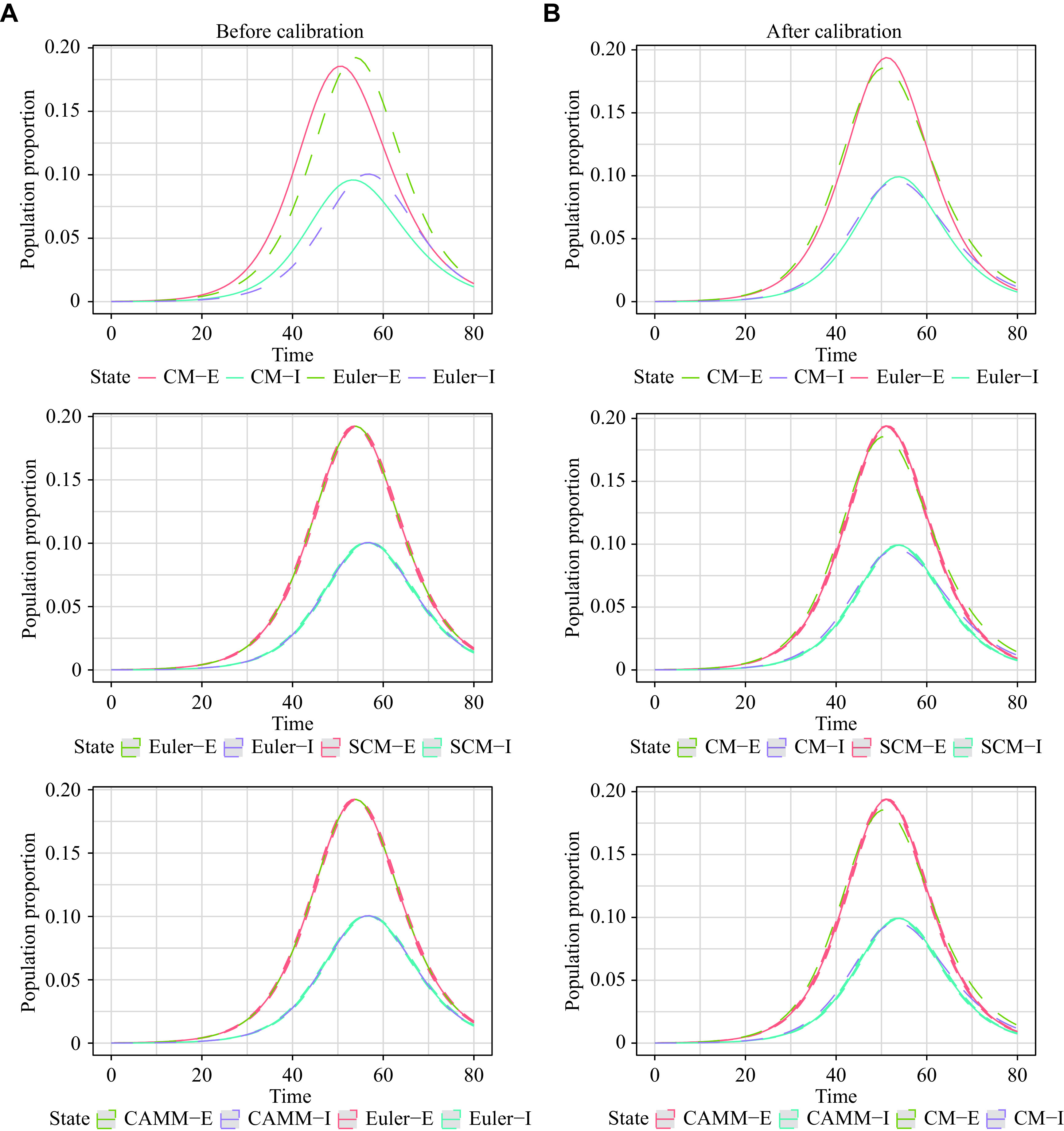
Population change curves of *E*, and *I* states of CM, SCM, and CAMM in scenario 2.

The results for scenario 2 were similar to those for scenario 1 (***Table 1***). RMSE_1_ and MAE_1_ for SCM and CAMM were small, showing the equivalence of the Euler method solutions, SCM, and CAMM. However, even after calibration, the difference between numerical solutions of CM by the LSODA and Euler methods still existed. Therefore, the exact solution of continuous-time CM cannot be perfectly reproduced by the corresponding discrete-time stochastic models. ***Fig. 3*** visualizes these results. 95% reference ranges were too narrow to be displayed in the figure because of the large population *N*.

### Scenario 3: SEIRD model

For scenario 3, the SEIRD model was constructed to describe the COVID-19 epidemic in Republic of Korea^[18]^. The initial values of SEIRD were $[ S(0),\;E(0),\;I(0),\;R(0),\;   D  (0) ] =   \left[ {9\,948,\;0,\;31,\;0,\;0} \right]$, and $N = 9\,979$ was the total number of the population without considering natural births and deaths of the population^[18]^. The derived parameters for CM, SCM, ABM, and CAMM were $\left( {\alpha, \;\beta,\; \gamma,\; \delta } \right) = ( 0.159\,6,\;   1/ 3{{.945\,7}},\;0.032\,8,\;0.000\,9 )$ and ${R_0} = \dfrac{{\alpha S \left( 0 \right)}}{{\left( {\gamma + \delta } \right)N}} = 4.721\,2$. The models of Euler, SCM, ABM, and CAMM were calibrated as described in the previous section, and the parameters after calibration were $\left( {\alpha ,\;\beta ,\;\gamma ,\;\delta } \right) =   \left( 0.164\,0,\;0.255\,3,\;0.033\,1,\; 0.001\,1 \right)$, with time span $\left[ {0,{\text{T}}} \right] = \left[ {0,150} \right]$.

**Figure 4:**
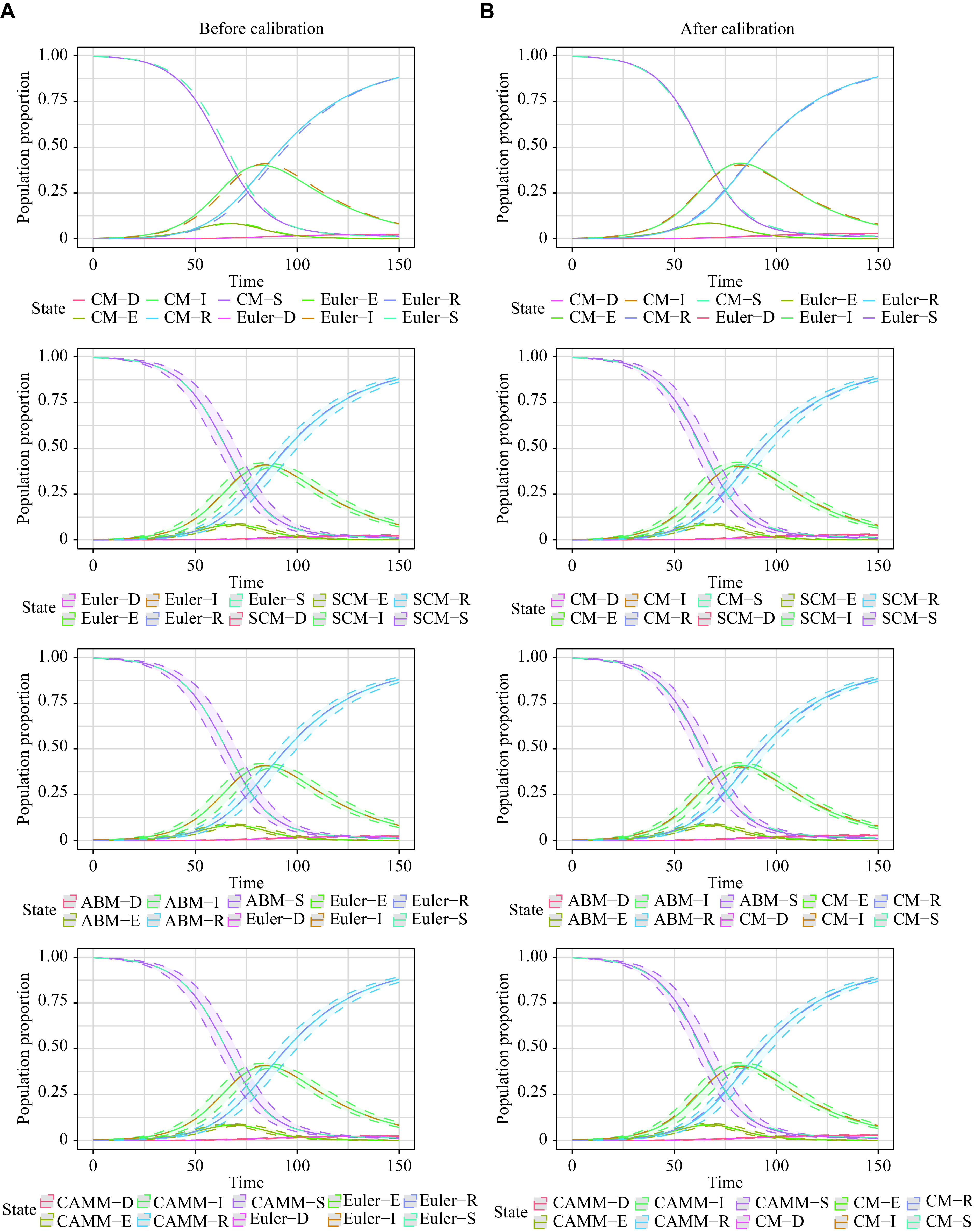
Population change curves of *S*, *E*, *I*, *R*, and *D* states of CM, SCM, ABM, and CAMM in scenario 3.

The results for scenario 3 were similar to those for scenario 2 (***Table 1***). RMSE_1_ and MAE_1_ showed the equivalence of the Euler method solutions, SCM, ABM, and CAMM. The difference between the exact solutions of CM by the LSODA and its stochastic models still existed after calibration. These results are shown in ***Fig. 4***. 95% reference ranges of stochastic models enclosed the curves of the CM numerical solutions.

### Scenario 4: SEIHR model

For scenario 4, the utilized SEIHR model was constructed to study the COVID-19 epidemic after the blockade in Wuhan^[19]^. Because of the large population base in the real data, ABM was difficult to handle, so we only demonstrated the equivalence between CM by the Euler method, SCM and CAMM. The total population was set to be $N = 10\,800\,000$. The initial values of the model were set to be $[ S(0), \;  E(0),\;   I(0),\;   H(0),\;R(0) ] = \left[ {10\,793\,032,\;4\,436,\;1\,914,\;533,\;85} \right]$ with time span $\left[ {0,T} \right] = \left[ {0,200} \right]$, and the parameters in CM, SCM, and CAMM were $(\beta ,\;\lambda ,\;\alpha ,\;\gamma ,\;\mu ) = (0.619\,5,    0.483\,5,\;0.160\,4,\;0.238\,9,\;0.042\,8)$ and $ {R_0} = \dfrac{{\beta S \left( 0 \right)}}{{\left( {\alpha + \gamma } \right)N}} =   1.550\,5 $^[19]^.

The models of Euler, SCM, and CAMM were calibrated as described in the previous section, and the parameters after calibration were $(\beta ,\;\lambda ,\;\alpha ,\;\gamma ,\;\mu ) =   (0.643\,6,\;0.507\,3,\;0.155\,3,\;0.260\,7,\;0.040\,4)$. CM solutions by the LSODA and Euler methods, corresponding SCM, and CAMM shared the same parameters. The simulation replication number for SCM and CAMM and the maximum number of agents for CAMM were set to 1000. RMSE and MAE were calculated as the RMSE and MAE for *E*, *I,* and *H* states' percentages of the initial population *N*.

**Figure 5:**
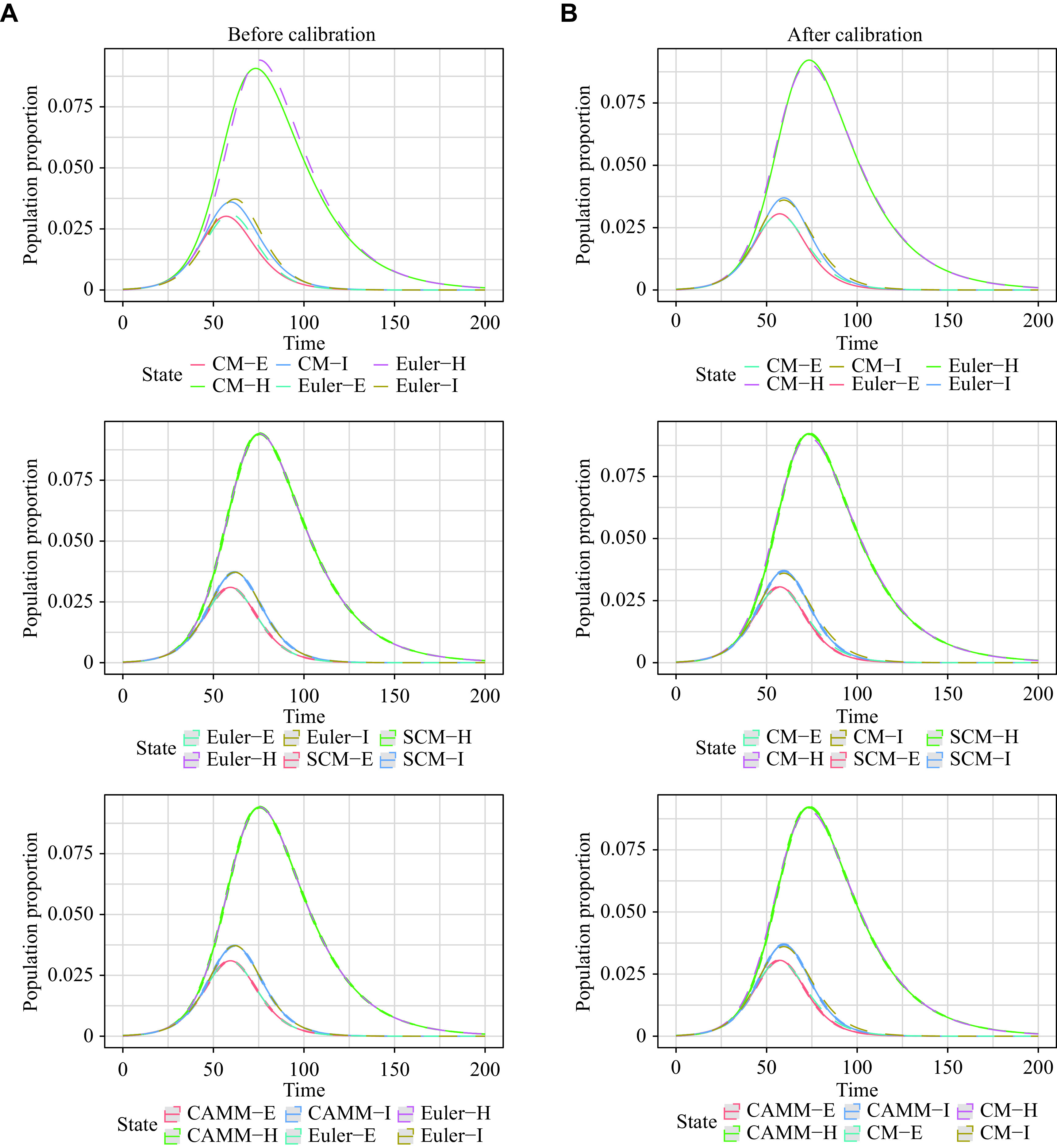
Population change curves of *E*, *I*, and *H* states of CM, SCM, and CAMM in scenario 4.

As presented in ***Table 1***, the disparities in RMSE_1_ and MAE_1_ among SCM, CAMM, and CM using the Euler method were negligible both before and after calibration in scenario 4. Hence, it was concluded that the CM by the Euler method was equivalent to both SCM and CAMM. However, the RSME_0_ and MAE_0_ of CM by the Euler method, SCM, and CAMM were relatively large, indicating that there were differences between them and CM by the LSODA method, even after the model calibration. Therefore, the numerical solutions of CM, obtained by the Euler method and its corresponding stochastic models, SCM and CAMM, cannot exactly simulate the CM exact solutions. ***Fig. 5*** is a visualization of these results. To make it clearer, *S* and *R* were hidden in the visualization, and the population proportions of *E*, *I*, and *H* states were shown.

## Discussion

Infectious disease modeling plays a crucial role in public health research^[11]^. While continuous-time deterministic CMs have long been the foundation of epidemic models, discrete-time stochastic models, such as SCMs and ABMs, have emerged to address some limitations of CMs^[46]^. Each modeling approach has its strengths and limitations. CMs are simpler and computationally less demanding, compared with stochastic models, but may lack the realism of more complex models because of the assumption of homogeneous populations within compartments. SCMs introduce randomness in inter-compartment transitions, while ABMs simulate individual-level interactions, providing highly detailed simulations. However, ABMs often require more computational resources and can be challenging to validate. Investigators should carefully choose the most appropriate model for their specific research problem, considering factors such as model assumptions, data availability, and computational resources.

The challenge in epidemiological studies lies in bridging the macroscopic and microscopic aspects. In the present study, we proposed a novel model, CAMM, which combined the macroscopic compartment of CM with the microscopic simulation of ABM. CAMM has integrated the advantages of both CM and ABM, and can serve as an alternative to ABM, when the number of simulation agents is limited. For instance, when simulating a large population, using ABM with one agent per person may become computationally intractable. In such cases, CAMM may offer a tractable simulation with a limited number of agents.

While CMs can be converted into corresponding stochastic counterparts including SCMs, ABMs, and CAMMs, it is important to note that the exact solutions of continuous-time CMs cannot perfectly match with discrete-time stochastic counterparts using the same parameter settings. The equivalence of CM numerical solutions using the Euler scheme, SCMs, ABMs, and CAMMs can be verified through existing theorems in the literature^[26]^. However, the Euler scheme is a basic numerical method for solving CMs, and its solutions differ from exact solutions and solutions obtained using more sophisticated and accurate schemes, such as LSODA and Runge-Kutta. We have demonstrated the differences between the exact CM solutions and solutions obtained from the four equivalent models with the same parameter settings. Therefore, caution should be exercised when calibrating stochastic models to reproduce the exact results of CMs. Direct model calibration of stochastic models can be time-consuming because of the need for averaging solutions from multiple simulation replications. To address this, we have proposed an efficient model calibration method based on CMs using the Euler scheme. This method minimizes the differences between the exact CM solutions and solutions obtained from stochastic methods, although slight discrepancies persist. It is important to note that discrete-time stochastic models cannot perfectly reproduce the exact solutions of continuous-time CMs.

Deterministic CMs are computationally efficient, but can only estimate the average values for each compartment. On the other hand, stochastic models are computationally less efficient, but because of the introduction of randomness, the interval estimates for each compartment can be calculated. Our findings can be applied to construct and compare deterministic CMs and corresponding stochastic models. This allows efficient model calibration of stochastic models, thereby creating a unified modeling framework that can be flexibly selected according to the practical application requirements of infectious disease prediction and control. Stochastic models with complex structures, such as ABMs, can be fully or partially converted to CMs, based on the equivalence between CMs using the Euler scheme and their corresponding stochastic models. Our proposed model calibration method enables efficient parameter estimation, improving the efficiency of stochastic model prediction. This, in turn, enhances the efficiency of comparing stochastic models and CMs. Furthermore, by bridging CMs and stochastic models under this unified framework, we have provided an efficient tool for HM construction and parameter estimation.

In conclusion, CMs are highly related to their stochastic counterparts (SCMs, ABMs, and CAMMs). We have verified the equivalence between CMs using the Euler scheme and their corresponding stochastic models. With limited computational resources, the proposed CAMM offers scalability and has the potential to serve as a substitute for ABM in simulating various infectious diseases in large-scale populations. Model calibration is necessary to reproduce the exact solutions of CMs using SCMs, ABMs, and CAMM. Here, we propose an efficient model calibration method based on the equivalence of these models, which can be extended to HMs. Our findings have contributed to the comparison and unification of deterministic CMs and stochastic models in the application of epidemic prediction and control.

## SUPPLEMENTARY DATA

Supplementary data to this article can be found online.

## SUPPLEMENTARY DATA

Supplementary data to this article can be found online.
