# Supplementary material for: The relationship between compartment models and their stochastic counterparts: A comparative study with examples of the COVID-19 epidemic modeling
Source: J Biomed Res. 2024 Mar 5;38(2):175–88. doi: 10.7555/JBR.37.20230137 (PMC11001592; doi:10.7555/JBR.37.20230137)
Supplement: Supplementary file 1 — Supplementary data to this article can be found online. [file jbr-38-2-175-S1.pdf]

Available online at www.jbr-pub.org.cn
Open Access at PubMed Central
Journal of Biomedical Research, 2024 0(000): 1–3
Supplementary Data
JBR

The relationship between compartment models and their stochastic counterparts: A comparative study with examples of the COVID-19 epidemic modeling

Ziyu Zhao[1,△], Yi Zhou[1,△], Jinxing Guan[1], Yan Yan[2], Jing Zhao[2], Zhihang Peng[1], Feng Chen[1,3], Yang Zhao[1,3,4,5,✉], Fang Shao[1,✉]

[1]*Department of Biostatistics, School of Public Health, Nanjing Medical University, Nanjing, Jiangsu 211166, China;*
[2]*Nanjing Hanwei Public Health Research Institute Co., Ltd, Nanjing, Jiangsu 210005, China;*
[3]*China International Cooperation Center for Environment and Human Health, Center for Global Health, Nanjing Medical University, Nanjing, Jiangsu 211166, China;*
[4]*The Center of Biomedical Big Data and the Laboratory of Biomedical Big Data, Nanjing Medical University, Nanjing, Jiangsu 211166, China;*
[5]*Jiangsu Key Lab of Cancer Biomarkers, Prevention and Treatment, Collaborative Innovation Center for Cancer Personalized Medicine, Nanjing Medical University, Nanjing, Jiangsu 211166, China.*

1 Corresponding stochastic models of SEIRD and SEIHR

1.1 Stochastic counterparts of SEIRD

The following discrete-time model is equivalent to the basic Euler numerical scheme for solving SEID model, where we set equally spaced discrete-time step $t \in \{0, 1, 2, \cdots, T-1\}$ with the time span $[0, T]$ for simplicity.

Based on CM, the corresponding SCM is as follows.

$$\begin{cases} \hat{S}_{t+1} = \hat{S}_t - s_t \\ \hat{E}_{t+1} = \hat{E}_t + s_t - e_t \\ \hat{I}_{t+1} = i_t + e_t \\ \hat{R}_{t+1} = \hat{R}_t + r_t \\ \hat{D}_{t+1} = \hat{D}_t + d_t \\ s_t \sim Binomial\left(\hat{S}_t, \dfrac{\alpha \hat{I}_t}{N}\right) \\ e_t \sim Binomial\left(\hat{E}_t, \beta\right) \\ (r_t, d_t, i_t) \sim Multinomial\left(\hat{I}_t, p\right) \end{cases} \tag{S1}$$

Here we use a multinomial random variable, $(r_t, d_t, i_t) \sim Multinomial\left(\hat{I}_t, p\right)$, with probabilities $p = (\gamma, \delta, 1-\gamma-\delta)$ to generate three new increments for the number of recoveries, the number of deaths, and the number of remaining infected.

The corresponding ABM is as follows.

△These authors contributed equally to this work.

✉Corresponding authors: Fang Shao and Yang Zhao, Department of Biostatistics, School of Public Health, Nanjing Medical University, 101 Longmian Avenue, Nanjing, Jiangsu 211166, China. E-mails: shaofang@njmu.edu.cn (Shao) and yzhao@njmu.edu.cn (Zhao).

Received: 11 June 2023; Revised: 01 September 2023; Accepted: 06 September 2023; Published online: 05 March 2024
CLC number: R181, Document code: A
The authors reported no conflict of interests.

This is an open access article under the Creative Commons Attribution (CC BY 4.0) license, which permits others to distribute, remix, adapt and build upon this work, for commercial use, provided the original work is properly cited.

© 2024 by Journal of Biomedical Research.
https://doi.org/10.7555/JBR.37.20230137

$$a_i(t+1) = \begin{cases} a_i(t) + Bernoulli\left(\frac{\alpha \hat{I}_t}{N}\right), & \text{if } a_i(t) = 1 \\ a_i(t) + Bernoulli(\beta), & \text{if } a_i(t) = 2 \\ a_i(t) + Index(n_t), & \text{if } a_i(t) = 3 \\ a_i(t), & \text{if } a_i(t) = 4 \text{ or } 5 \end{cases} \quad \text{(S2)}$$

Here, $a_i(t)$is the agent $i \in \{1, 2, \cdots, N\}$ at the time $t \in \{0, 1, \cdots, T\}$, and the values of $a_i(t)$, 1, 2, 3, 4, and 5 refer to the S, E, I, R, and D compartment states, respectively, and $\hat{I}_t$ is the number of agents with state I at the time t. The above multinomial random variable, $n_t = (r_t, d_t, i_t) \sim Multinomial(1, p)$, follows a multinomial distribution with probabilities $p = (\gamma, \delta, 1-\gamma-\delta)$ for recovered, dead, and continued infected states, respectively. The $Index(\cdot)$ function gives the value 1 if $r_t = 1$, 2 if $d_t = 1$, and 0 if $i_t = 1$.

The corresponding CAMM is as follows.

For the time step $t = 0$, the initial step is performed as follows.

Step 0. Generate only five agents, $a_{i,0}$, where $State(a_{i,0}) = i$and $Size(a_{i,0})$ is the i-th element of $[S(0), E(0), I(0), R(0), D(0)]$ for $i \in \{1, 2, 3, 4, 5\}$. Here, the values of $State(a_{i,0})$, 1, 2, 3, 4, and 5, refer to the S, E, I, R, D compartment states, respectively. $Size(a_{i,0})$ is the size of the compartment agent with nonnegative integer values, which stands for the number of cases for the agent.

For each discrete-time step $t \in \{0, 1, 2, \cdots, T-1\}$, the following two steps are performed.

Step 1. For each agent $a_{i,t}$ at a time step t, we do the followings.

a. If $State(a_{i,t}) \leqslant 2$, $State(a_{i,t+1}) = State(a_{i,t})$ and $Size(a_{i,t}+1) = Size(a_{i,t}) - as_{i,t}$, where $Sia_{i,t} = Size(a_{i,t})$ and

$$as_{i,t} \sim \begin{cases} Binomial\left(Sia_{i,t}, \frac{\alpha \hat{I}_t}{N}\right), & if\ State(a_{i,t}) = 1 \\ Binomial(Sia_{i,t}, \beta), & if\ State(a_{i,t}) = 2 \end{cases}.$$

Next, a new agent $a_{new\_i,t+1}$ is generated with $State(a_{new\_i,t+1})$ =$State(a_{i,t}) + 1$ and $Size(a_{new\_i,t+1}) = as_{i,t}$.

b. If $State(a_{i,t}) = 3$, $State(a_{i,t+1}) = 3$ and $Size(a_{i,t+1}) = i_t$, where $(r_t, d_t, i_t) \sim Multinomial(Sia_{i,t}, p)$, $Sia_{i,t} = Size(a_{i,t})$ and $p = (\gamma, \delta, 1-\gamma-\delta)$.

Next, two new agents $a_{new\_i\_R,t+1}$ and $a_{new\_i\_D,t+1}$ are generated with

$$State(a_{new\_i\_R,t+1}) = 4, Size(a_{new\_i\_R,t+1}) = r_t,$$

$$State(a_{new\_i\_D,t+1}) = 5, \text{ and } Size(a_{new\_i\_D,t+1}) = d_t.$$

c. If $State(a_{i,t}) > 3$, $State(a_{i,t+1}) = State(a_{i,t})$ and $Size(a_{i,t+1}) = Size(a_{i,t})$.

Here, the values of $State(a_{i,t})$, 1, 2, 3, 4 and 5, refer to the S, E, I, R, and D compartment states, respectively. $Size(a_{i,t})$ is the size of the compartment agent with nonnegative integer values. And $\hat{I}_t = \sum_{State(a_{j,t})=3} Size(a_{j,t})$ is the total number of individuals with state I at the time t.

Step 2. If the total number of agents at time step $t+1$ exceeds the predefined maximum number of agents, then agents are concatenated to S, E, I, R, D compartments as follows. For $i \in \{1, 2, 3, 4, 5\}$, $State(a_{i,t+1}) = i$ and $Size(a_{i,t+1}) = \sum_{Size(a_{j,t+1})=i} Size(a_{j,t+1})$.

Remove all other agents $a_{i,t+1}$ for $i \notin \{1, 2, 3, 4, 5\}$.

1.2 Stochastic counterparts of SEIHR

The following discrete-time model is equivalent to the basic Euler numerical scheme for solving SEIHR model, where we set equally spaced discrete time step $t \in \{0, 1, 2, \cdots, T-1\}$ with the time span $[0, T]$ for simplicity.

Based on CM, the corresponding SCM is as follows.

$$\begin{cases} \hat{S}_{t+1} = \hat{S}_t - s_t \\ \hat{E}_{t+1} = \hat{E}_t + s_t - e_t \\ \hat{I}_{t+1} = i_{i,t} - e_t \\ \hat{H}_{t+1} = \hat{H}_t + i_{H,t} - h_t \\ \hat{R}_{t+1} = \hat{R}_t + i_{R,t} + h_t \\ s_t \sim Binomial\left(\hat{S}_t, ps_t\right) \\ e_t \sim Binomial\left(\hat{E}_t, \lambda\right) \\ (i_{H,t}, i_{R,t}, i_{i,t}) \sim Multimonial\left(\hat{I}_t, p\right) \\ h_t \sim Binomial\left(\hat{H}_t, \mu\right) \end{cases} \quad \text{(S3)}$$

Where $ps_t = \beta\left(1 - \frac{\hat{I}_t}{N}\right)\frac{\hat{I}_t}{N}$, and we use a multinomial random variable, $(i_{H,t}, i_{R,t}, i_{i,t}) \sim Multimonial\left(\hat{I}_t, p\right)$, with probabilities $p = (\alpha, \gamma, 1-\alpha-\gamma)$ to generate three new increments for the number of hospitalizations, the number of removals, and the number of remaining infections, respectively.

Corresponding ABM is derived as follows.

$$a_i(t+1) = \begin{cases} a_i(t) + Bernoulli(ps_t), & \text{if } a_i(t) = 1 \\ a_i(t) + Bernoulli(\lambda), & \text{if } a_i(t) = 2 \\ a_i(t) + Index(n_t), & \text{if } a_i(t) = 3 \\ a_i(t) + Bernoulli(\mu), & \text{if } a_i(t) = 4 \\ a_i(t), & \text{if } a_i(t) = 5 \end{cases} \quad \text{(S4)}$$

Here, $a_i(t)$is the agent $i \in \{1, 2, \cdots, N\}$ at the time $t \in \{0, 1, \cdots, T\}$, and the values of $a_i(t)$, 1, 2, 3, 4, and 5 refer to the S, E, I, H, and R compartment states, respectively, and $\hat{I}_t$ is the number of agents with state I

at time t. $ps_t = \beta\left(1 - \frac{\hat{I}_t}{N}\right)\frac{\hat{I}_t}{N}$. The above multinomial random variable, $n_t = (h_t, r_t, i_t) \sim Multinomial(1, p)$, follows a multinomial distribution with probabilities $p = (\alpha, \gamma, 1 - \alpha - \gamma)$ for recovered, dead, and continued infected states, respectively. The $Index(\cdot)$ function gives the value 1 if $h_t = 1$, 2 if $r_t = 1$, and 0 if $i_t = 1$.

Regarding CAMM, the algorithm is shown as follows.

For the time step $t = 0$, the initial step is performed as follows.

Step 0. Generate only four agents, $a_{i,0}$, where $State(a_{i,0}) = i$ and $Size(a_{i,0})$ is the i-th element of $[S(0), E(0), I(0), H(0), R(0)]$ for $i \in \{1, 2, 3, 4, 5\}$. Here, the values of $State(a_{i,0})$, 1, 2, 3, 4 and 5, refer to the S, E, I, H, and R compartment states, respectively. $Size(a_{i,0})$ is the size of the compartment agent with nonnegative integer values.

For each discrete time step $t \in \{0, 1, 2, \ldots, T-1\}$, the following two steps are performed.

Step 1. For each agent $a_{i,t}$ at a time step t, do the followings.

a. If $State(a_{i,t}) \in \{1, 2, 4\}$, $State(a_{i,t+1}) = State(a_{i,t})$, $Size(a_{i,t+1}) = Size(a_{i,t}) - as_{i,t}$, where $Sia_{i,t} = Size(a_{i,t})$, $ps_t = \beta\left(1 - \frac{\hat{I}_t}{N}\right)\frac{\hat{I}_t}{N}$ and

$$as_{i,t} \sim \begin{cases} Binomial(Sia_{i,t}, ps_t), & \text{if } State(a_{i,t}) = 1 \\ Binomial(Sia_{i,t}, \lambda), & \text{if } State(a_{i,t}) = 2 \\ Binomial(Sia_{i,t}, \mu), & \text{if } State(a_{i,t}) = 4 \end{cases} \tag{S5}$$

Next, a new agent $a_{new\_i,t+1}$ is generated with $State(a_{new\_i,t+1}) = State(a_{i,t}) + 1$ and $Size(a_{new\_i,t+1}) = as_{i,t}$.

b. If $State(a_{i,t}) = 3$, $State(a_{i,t+1}) = 3$ and $Size(a_{i,t+1}) = i_t$, where $(h_t, r_t, i_t) \sim Multinomial(Sia_{i,t}, p)$, $Sia_{i,t} = Size(a_{i,t})$ and $p = (\alpha, \gamma, 1 - \alpha - \gamma)$.

Next, two new agents $a_{new\_i\_r,t+1}$ and $a_{new\_i\_d,t+1}$ are generated with $State(a_{new\_i\_H,t+1}) = 4$, $Size(a_{new\_i\_H,t+1}) = h_t$, $State(a_{new\_i\_R,t+1}) = 5$, and $Size(a_{new\_i\_R,t+1}) = r_t$.

c. If $State(a_{i,t}) = 5$, $State(a_{i,t+1}) = State(a_{i,t})$ and $Size(a_{i,t+1}) = Size(a_{i,t})$.

Here, the values of $State(a_{i,t})$, 1, 2, 3, 4 and 5, refer to the S, E, I, H, and R compartment states, respectively. $Size(a_{i,t})$ is the size of the compartment agent with nonnegative integer values. And $\hat{I}_t = \sum_{State(a_{j,t})=3} Size(a_{j,t})$ is the total number of individuals with state I at the time t.

Step 2. If the total number of agents at the time step $t+1$ exceeds the predefined maximum number of agents, then concatenate agents to S, E, I, H, and R compartments as follows.

For $i \in \{1, 2, 3, 4, 5\}$, $State(a_{i,t+1}) = i$ and $Size(a_{i,t+1}) = \sum_{State(a_{j,t+1})=i} Size(a_{j,t+1})$.

Remove all other agents $a_{i,t+1}$ for $i \notin \{1, 2, 3, 4, 5\}$.

2 Code availability

Sample codes for simulations and analyses in the current study are provided as a separate compressed file (***R_code.rar***, available online).
